# Methodological Advances in Mitochondrial DNA Analysis for Forensic Genetics

**DOI:** 10.3390/genes17060609

**Published:** 2026-05-28

**Authors:** Víctor Daniel Carrillo-Rodríguez, Carina Amalinalli Ruiz-Villavicencio, María Teresa Navarro-Romero, Héctor Rangel-Villalobos, Cecilia Martínez-Campos

**Affiliations:** 1 Laboratorio de Investigación e Innovación en Genética Forense (LIIGF), Instituto Nacional de Medicina Genómica (INMEGEN), Mexico City 14610, Mexico; victor.carrillo1004@alumnos.udg.mx (V.D.C.-R.); aruiz@inmegen.gob.mx (C.A.R.-V.); mnavarro@inmegen.edu.mx (M.T.N.-R.); 2 Centro Universitario de Ciencias de la Salud, Universidad de Guadalajara, Guadalajara 44340, Jalisco, Mexico; 3 Instituto de Investigación en Genética Molecular, Centro Universitario de la Ciénega, Universidad de Guadalajara (CUCI-UdeG), Ocotlán 47820, Jalisco, Mexico; hector.rangel@academicos.udg.mx

**Keywords:** mitochondrial DNA, forensic genetics, massive parallel sequencing, third-generation sequencing (TGS), human identification

## Abstract

Mitochondrial DNA (mtDNA) analysis is a fundamental tool in forensic genetics, particularly when biological samples exhibit severe degradation or low nuclear DNA content. Its unique biological characteristics, such as a high copy number per cell, strict matrilineal inheritance, and lack of recombination, enable human identification and reconstruction of maternal lineages in complex contexts, including disaster victim identification, historical cases, and missing persons investigations. This narrative review examines contemporary methodological approaches for investigating the human mitogenome. We discuss recent advancements in extraction and enrichment techniques, emphasizing their efficacy in reducing the interference of nuclear mitochondrial DNA sequences (NUMTs) and enhancing the recovery of informative fragments. Moreover, the shift from traditional Sanger sequencing to Massive Parallel Sequencing (MPS) is examined, as MPS has markedly enhanced the sensitivity and capability of contemporary methods to detect low-frequency heteroplasmies. Additionally, the advent of Third-Generation Sequencing (TGS), exemplified by nanopore platforms, is evaluated, which facilitates the reading of full-length native molecules without the biases introduced by PCR amplification. Despite the interpretive challenges posed by heteroplasmy, contamination, and limitations in population databases, ongoing methodological advances in mitochondrial DNA analysis continue to strengthen its reliability and expand its potential in forensic genetics.

## 1. Introduction

Human mitochondrial DNA (mtDNA) is a compact circular molecule containing 16,569 base pairs (bp) and accounts for approximately 1/100,000 of the haploid human genome [1]. It consists of two strands: a guanine-rich heavy (H) strand and a complementary light (L) strand, each with its own origin of replication (OH and OL, respectively) [2]. mtDNA is primarily inherited through the maternal lineage, implying cellular processes to eliminate paternally derived mitochondria post-fertilization, thereby avoiding inter-genomic competition [3,4]. The proposed mechanisms underlying the strict maternal inheritance of mtDNA include the dilution of sperm-derived mitochondria in the oocyte cytoplasm, selective ubiquitination and autophagic degradation of paternal mitochondria, and mitochondrial genetic bottlenecks that limit paternal transmission [5].

The absence of recombination, combined with uniparental inheritance, enables mtDNA to maintain lineage-specific variations across evolutionary timescales. This characteristic facilitates the reconstruction of maternal ancestry, akin to the inference of paternal ancestry through the non-recombining region of the Y chromosome [3]. Reports of paternal mtDNA transmission have emerged from clinical and multigenerational studies [6,7]. These findings remain controversial, as some authors attribute them to methodological artifacts, such as contamination or amplification of nuclear mitochondrial DNA sequences (NUMTs), rather than to true paternal inheritance [8,9].

Owing to its high copy number and unique biological and evolutionary characteristics, mtDNA has become integral to forensic genetics, particularly in cases involving highly degraded samples and limited biological material. Its pattern of maternal inheritance and relative stability in compromised specimens render it especially valuable for human identification, investigations of missing persons, disaster victim identification, and the analysis of historical or ancient remains. In this context, the present review aims to examine the current methodological approaches for human mitochondrial genome analysis in forensic science, with particular emphasis on advances in extraction and enrichment strategies and sequencing technologies. Special attention has been given to the transition from conventional Sanger sequencing to massively parallel sequencing (MPS) and emerging third-generation sequencing (TGS) technologies, which have substantially improved sensitivity, heteroplasmy detection, and complete mitogenome recovery. By integrating recent methodological developments and discussing their limitations and forensic applicability, this review highlights the evolving role of mtDNA analysis as an increasingly powerful and reliable tool in forensic genetics.

## 2. Genetic Variants Within mtDNA and Nomenclature

Sequence variations arise from mutations, which occur at a higher rate in mtDNA than in nuclear DNA, largely due to their proximity to the electron transport chain and the resulting exposure to reactive oxygen species (ROS) generated during oxidative phosphorylation (OXPHOS) [1,4,10]. These mutations predominantly comprise single-nucleotide variants (SNVs) and insertion–deletion events (INDELs), which contribute to the emergence of heteroplasmy, the coexistence of multiple mtDNA sequence variants within an individual. This condition typically arises from point mutations or length variations and is often observed at higher levels in tissues with high metabolic demands, such as the brain, liver, and skeletal muscles [1]. A set of mtDNA variants inherited together as a single, non-recombining unit defines a mitochondrial haplotype [11].

The non-coding region (also known as the Control Region) encompasses three hypervariable regions (HVR-I, HVR-II, and HVR-III), which offer the highest discriminatory power for human identification (HID) and therefore represent the primary targets in forensic mtDNA analysis [10,12,13,14,15]. Closely related haplotypes cluster into haplogroups that reflect shared maternal ancestry and often display distinct geographic distributions, enabling phylogeographic reconstructions of human maternal dispersal [3,14,16].

The revised Cambridge Reference Sequence (rCRS) serves as an international standard for reporting human mtDNA variations in forensic, medical, and population genetics research. First published as the Cambridge Reference Sequence (CRS) in 1981, it was revised in 1999 to correct 11 sequencing errors, resulting in the rCRS [17,18]. This framework enables concise and standardized reporting by documenting only the nucleotide positions that differ from the reference sequence. Moreover, its availability in public databases, such as GenBank, ensures data accessibility, facilitates inter-laboratory comparability, and promotes consistency across studies [19].

Although the Reconstructed Sapiens Reference Sequence (RSRS) was proposed as an alternative mtDNA reference framework to better distinguish between ancestral and derived alleles through the incorporation of thousands of modern and archaic mitogenomes, the revised Cambridge Reference Sequence (rCRS) remains the standard in forensic genetics because of its extensive validation and widespread adoption [19,20].

In forensic practice, mitochondrial profiles are defined by a set of polymorphisms that differentiate a sample from the rCRS and are typically reported using circumfix notation, in which only variant positions relative to the reference sequence are listed [11,21,22]. Interpretation is particularly challenging in the homopolymeric regions of the control region, where length heteroplasmy is common; consequently, current guidelines recommend haplogroup-informed multiple-sequence alignment to ensure an accurate representation of complex variants [23].

## 3. Forensic Applications of Mitochondrial DNA Analysis

**Figure 1:**
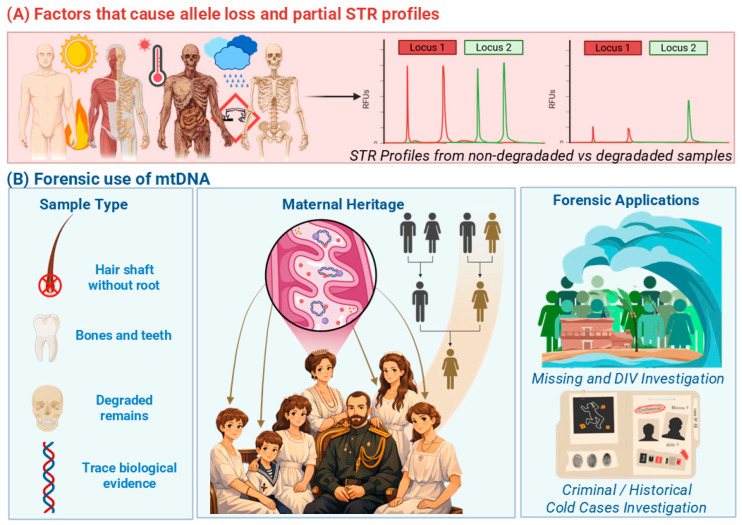
Forensic Applications of Mitochondrial DNA in Challenging Samples: Schematic overview illustrating the forensic utility of mtDNA when nuclear DNA is limited or degraded. (**A**) Environmental and post-mortem factors, such as temperature, humidity, and weathering, can degrade DNA and lead to allele dropout or partial short tandem repeat (STR) profiles. The panel contrasts complete STR profiles obtained from well-preserved samples with partial or incomplete profiles obtained from degraded materials. (**B**) In such contexts, mtDNA analysis provides a valuable alternative because of its high copy number per cell and greater persistence in degraded tissues. mtDNA can be successfully recovered from challenging sample types, including hair shafts, bones, teeth, and highly degraded human remains. Because mtDNA is maternally inherited, it enables comparisons with maternal relatives and is widely applied in forensic investigations, including missing person identification, disaster victim identification (DVI), criminal casework, and historical or cold-case investigations. Figure created with the assistance of artificial intelligence (AI)-based visualization tools and BioRender.com; all scientific content and methodological interpretations were reviewed and verified by the authors.

In forensic genetics, mtDNA analysis serves as a valuable complementary tool for the examination of degraded or low-template biological samples that are unsuitable for conventional autosomal short tandem repeat (STR) profiling. In this context, mtDNA can often be recovered from challenging forensic substrates, including rootless hair shafts, bones, teeth, highly degraded remains, and trace biological evidence [4,11,12,13,14]. Consequently, mtDNA analysis has become an important tool in kinship testing, unidentified human remains investigations, and the examination of historical or cold cases [24,25,26] (Figure 1). While mtDNA offers less individual discriminatory power than nuclear STR markers, mtDNA haplotypes can still yield substantial associative evidence when analyzed within phylogenetic and population-genetic frameworks [19].

The forensic utility of mtDNA for human identification was first demonstrated in the late 1980s and the early 1990s through pioneering studies involving highly degraded human remains [15]. Stoneking et al. analyzed the HVI and HVII regions of mtDNA from the skeletal remains of a child and demonstrated that mtDNA was consistent with the corresponding maternal reference sample. This study provided one of the earliest demonstrations of the feasibility of mtDNA analysis for human identification in degraded skeletal material, establishing a foundation for its subsequent application in forensic genetics [27]. The following year, Sullivan et al. demonstrated the value of mtDNA analysis for the identification of highly decomposed human remains through the successful characterization of mitochondrial sequences from skeletal and soft tissue [28]. This was followed by the landmark identification of the Romanov family remains by Peter Gill and collaborators, where mtDNA analysis confirmed maternal lineage relationships and provided compelling evidence supporting the identification of the remains [29].

The application of mtDNA analysis has rapidly expanded to contexts involving human rights violations, as demonstrated by Boles [30] and Corach [31], who identified victims of military repression in Guatemala and the Argentine dictatorship. The potential of mtDNA for identifying victims of mass disasters was highlighted after the 2004 Southeast Asian tsunami, which contributed to a high rate of successful identification [32]. Additional notable applications include the historical identification of King Richard III from England [33] and the identification of victims of the Spanish Civil War under highly complex forensic conditions [34].

Recent studies have incorporated next-generation sequencing (NGS) technologies for mtDNA analysis, as exemplified by Loreille et al. [35], who analyzed rootless hairs associated with the Romanov family and reported the detection of mitochondrial heteroplasmy across multiple generations, further strengthening the genetic evidence supporting the identification and maternal lineage relationships of the remains.

Collectively, these studies underscore the value of mtDNA as a robust and complementary tool in modern forensic genetics, with applications ranging from individual identification to the reconstruction of complex historical and humanitarian events. In this context, the efficient recovery of mtDNA from challenging substrates remains a critical determinant of successful analysis. The following sections examine the methodological advances in mitochondrial enrichment, extraction, and sequencing approaches that have been developed or hold promise for forensic applications.

## 4. DNA Extraction in Forensics

Although DNA extraction protocols differ in methodology, most follow a common workflow involving cell lysis, protein removal, DNA purification, washing, and final elution or resuspension [36,37]. In forensic, anthropological, and ancient DNA research, the selection of an extraction strategy is a critical analytical step because it directly influences the DNA yield, purity, fragment size recovery, and suitability for downstream analyses [35,38,39,40,41,42,43,44,45]. Accordingly, specialized protocols have been developed to address the challenges posed by highly degraded or limited biological material, particularly skeletal remains, burned tissues, and hair shafts [38,46,47,48,49]. Early studies established the methodological foundations for recovering DNA from archaeological and ancient specimens, demonstrating the feasibility of isolating fragmented genetic material from compromised samples [50,51,52]. More recent approaches, especially those adapted from ancient DNA research, prioritize the recovery of ultrashort DNA fragments and the reduction in inhibitory substances [39,40,42]. For example, Xavier et al. [42] compared the extraction methods developed by Dabney [39,40] and Loreille [35,41], and observed that the Dabney protocol produced a higher relative DNA yield and recovered a greater proportion of informative fragments shorter than 35 bp when normalized to the amount of processed bone powder. In contrast, the Loreille method tolerated larger quantities of starting material and generated a higher absolute DNA yield, making it more suitable when sample preservation is less compromised. Collectively, these studies demonstrate that extraction efficiency depends not only on the protocol itself but also on the preservation state of the sample and the specific analytical objectives, including mitochondrial and nuclear DNA recovery for forensic identification and genomic analysis [47,49,53].

Current forensic and ancient DNA workflows are generally based on four major methodological strategies: organic extraction methods, such as phenol–chloroform protocols; silica- or magnetic bead-based extraction systems; and hybrid workflows that combine elements of both strategies to optimize DNA recovery, purity, and fragment retention from degraded biological material. A summary of these approaches is presented in Table 1.

These comparative studies highlight how methodological choices during DNA recovery can substantially influence the quantity, quality, and analytical utility of genetic material obtained from ancient and forensic samples.

## 5. Mitochondrial DNA Enrichment

Genomic DNA typically comprises a mixture of nuclear and mitochondrial DNA. Therefore, forensic mtDNA analysis can benefit from enrichment strategies applied before and after extraction. Each of these approaches is based on different biochemical and molecular principles and can significantly influence DNA yield, molecular integrity, and downstream analytical performance.

### 5.1. Organelle- and Extraction-Based Enrichment Approaches 

Mitochondrial organelle isolation encompasses a range of methodologies designed to enrich intact mitochondria before downstream molecular analysis. In addition to conventional differential centrifugation, other strategies, such as density gradient fractionation with sucrose or Percoll [58,59,60,61] and immunoaffinity-based capture targeting outer membrane proteins such as TOMM22 [62,63], have been developed to improve mitochondrial DNA purity. Recently, nanobiopsy combined with next-generation sequencing has enabled subcellular mtDNA heteroplasmy analyses [54].

Selective mtDNA extraction workflows have also been incorporated into commercially available systems designed for use with fresh cells and tissues. These methods generally involve selective lysis and mechanical disruption to preserve mitochondrial integrity, followed by differential centrifugation and organelle lysis to recover purified mtDNA [57,64,65]. These approaches have been widely applied in studies investigating mitochondrial function across diverse biological contexts [66,67]. However, despite their effectiveness in enriching high-quality mtDNA, these methodologies depend on the preservation of intact cells and mitochondria, thereby limiting their applicability to fresh or well-preserved tissues. Consequently, their utility in forensic casework involving highly degraded samples remains largely unknown.

Selective enrichment strategies have focused on exploiting the structural properties of mtDNA. Quispe-Tintaya et al. [55] demonstrated that plasmid miniprep-based workflows can preferentially enrich mtDNA over nuclear DNA by leveraging the circular and supercoiled nature of the mitochondrial genome. When combined with magnetic bead purification, this approach achieved enrichment levels exceeding 2000-fold relative to the total genomic DNA [55].

### 5.2. Amplification-Based Enrichment Approaches

PCR-based enrichment continues to be extensively utilized, employing overlapping amplicon strategies to selectively amplify mtDNA. Early applications of this strategy consisted of coupling PCR with other techniques to enable the physical mapping of specific chromosomal regions, allowing the construction of maps around pericentromeric areas, polymorphic loci, and translocation breakpoints, facilitating gene localization and linkage analysis [68,69,70]. More recently, they have been adapted for the assessment of mtDNA damage [71].

Over time, different strategies have been proposed to efficiently amplify the entire mitochondrial genome using different numbers and configurations of primer pairs, demonstrating that mtDNA can be recovered through diverse amplification designs that generate amplicons suitable for downstream analyses [72,73,74,75]. These approaches have proven particularly valuable for forensic applications involving degraded skeletal remains, hair shafts, and other low-template biological materials, enabling the generation of high-quality mitochondrial control regions and whole-mitogenome sequences from compromised samples [76,77]. Chaitanya et al. developed a PCR-based mtDNA enrichment strategy using 161 short overlapping amplicons (~200 bp) to amplify the complete mitochondrial genome from degraded samples. By targeting short mtDNA fragments (~200 bp), this approach improves amplification success in fragmented and low-template materials compared with conventional long-range PCR methods. This study demonstrated high mitogenome coverage in degraded samples, highlighting the forensic utility of overlapping PCR enrichment strategies for compromised biological evidence [78]. Similarly, Zavala et al. applied ancient DNA-inspired short-fragment enrichment and sequencing methods to skeletal remains from Korean War and World War II unknowns, substantially improving mitochondrial and nuclear DNA recovery compared with conventional forensic workflows. Emery et al. reconstructed mitochondrial and nuclear genetic profiles from severely burned human remains using extraction and enrichment methods adapted from ancient DNA research, highlighting the effectiveness of short-amplicon and degraded DNA-focused workflows in disaster victim identification and highly compromised forensic specimens [48,49].

In contrast, long-range PCR enables the amplification of large mitochondrial DNA fragments, typically ranging from 12 to 15 kb, thereby reducing the number of primer pairs required for complete mitogenome coverage [79,80]. This strategy is particularly advantageous for high-quality DNA samples because it minimizes amplification bias, reduces the risk of contamination associated with multiple PCR reactions, and simplifies the downstream library preparation and sequence assembly. For example, Sukser et al. [76,77] demonstrated the successful amplification of the entire mitochondrial genome using two overlapping amplicons of approximately 9.1 and 11.2 kb. Using the Illumina^®^ (San Diego, CA, USA) Human mtDNA Genome assay on the MiSeq FGx™ platform, the authors established performance evaluation guidelines for forensic applications and demonstrated that the method produced reproducible and high-quality mitogenome sequences from buccal swabs and blood samples placed on FTA cards. Importantly, the resulting haplotypes were concordant with conventional Sanger sequencing data, supporting the reliability of long-range PCR for forensic mitogenome analysis of well-preserved samples [79]. However, the effectiveness of this approach is strongly dependent on DNA integrity, as highly degraded samples or specimens containing large mitochondrial deletions may not support the amplification of long continuous fragments. Consequently, long-range PCR is generally unsuitable for severely degraded forensic material, where short overlapping amplicon strategies provide greater amplification success and sequence recovery [68,79,80].

Unlike long-range PCR, which requires relatively intact DNA to amplify large fragments, primer extension capture (PEC) employs multiple short targets, making it particularly suitable for degraded or low-template samples commonly encountered in forensic and ancient DNA analyses. Originally developed for Neanderthal mitogenome studies [81], PEC was subsequently adapted for forensic mtDNA analysis [82]. Eduardoff et al. [82] optimized mtDNA control-region PEC analysis for highly compromised forensic samples of different ages and origins, demonstrating improved sensitivity and sequence recovery from degraded materials. Subsequently, Parson et al. [83] successfully applied PEC-MPS to Late Bronze Age skeletal remains from Stillfried, Austria, obtaining interpretable mitotypes that supported previously proposed matrilineal relationships. In forensic genetics, this approach has garnered increasing interest over the last decade, especially with its efficacy demonstrated in the analysis of mtDNA [82,84,85,86] and single-nucleotide polymorphisms [85,87,88,89] for kinship and identity determination.

#### Limitations of Amplification-Based Enrichment Approaches

Despite their high analytical sensitivity, amplification-based mtDNA enrichment strategies have important limitations that may compromise forensic interpretation. PCR-dependent approaches are particularly susceptible to amplification bias, allele dropout, and preferential amplification, especially when heteroplasmy or sequence polymorphisms occur within the primer-binding regions [11,90,91]. These factors can distort the variant representation and reduce the accuracy of heteroplasmy detection. In addition, PCR-based methods have limited ability to accurately characterize large-scale mitochondrial deletions, including precise breakpoint resolution and heteroplasmy quantification [92].

The fragmented nature of forensic DNA further restricts the applicability of long-amplicon strategies, particularly in highly degraded specimens such as skeletal remains, where extensive DNA fragmentation may prevent the successful amplification of large mtDNA regions despite partial molecular preservation within the biological matrix. Furthermore, while the biological matrix of tissue (particularly bone) may offer some protection to mtDNA against degradation, this same factor can render the long-amplicon approach unsuitable for a substantial proportion of forensic samples [93]. By targeting intermediate-sized fragments rather than a single long amplicon, these approaches improve amplification success and mitogenome recovery in degraded or mixed forensic samples while maintaining a broad genomic coverage.

### 5.3. Hybridization-Based Enrichment Approaches

Hybridization-based capture techniques have emerged as powerful enrichment strategies for mitochondrial and nuclear DNA analysis in forensic and ancient DNA research. These approaches rely on the hybridization of fragmented target DNA molecules to complementary biotinylated DNA or RNA probes (“baits”), followed by the selective isolation of probe-target complexes using streptavidin-coated magnetic beads [94,95]. By enabling the targeted recovery of specific genomic regions from highly fragmented DNA, hybridization capture substantially improves the proportion of informative sequences obtained during MPS while reducing background DNA and minimizing interference from nuclear mitochondrial DNA segments (NUMTs) [96,97,98].

A notable forensic application was reported by Marshall et al. [99], who employed custom hybridization probes together with MPS to enrich the complete mitochondrial genomes from 14 degraded long bones recovered from a mass grave in Croatia. This approach successfully generated complete mitogenomes from 12 samples, enabling the regrouping of commingled remains according to mitochondrial haplotypes and supporting the identification of sisters Marija and Tereza Kozulić through kinship analysis [99]. In addition, hybridization capture of shotgun sequencing libraries, rather than conventional PCR amplification, has demonstrated improved recovery of mtDNA from highly fragmented DNA while simultaneously enabling the analysis of complete mitochondrial genomes and nuclear SNP markers in challenging forensic samples [85].

#### Limitations of Hybridization-Based Enrichment Approaches

Despite its high sensitivity, hybridization capture is limited by suboptimal enrichment efficiency in samples with low endogenous DNA content. This approach also entails considerable analytical and economic costs, as it often produces high levels of PCR duplicates and off-target hybridization [96,97]. Consequently, the process often becomes a stochastic effort to “fish” for informative fragments within a vast metagenomic background, where a disproportionate amount of sequencing data must be discarded because of molecular redundancy and non-specific binding [98]. This lack of cost-effectiveness relegates this technique to a specialized last resort for highly degraded skeletal remains analysis.

### 5.4. Enzyme-Based Enrichment Approaches

Enzymatic techniques have proven effective for enriching mtDNA content in genomic extracts. These methods utilize enzymes to selectively eliminate nuclear DNA or target specific mitochondrial components. A commonly used technique involves the use of exonuclease V (ExoV), which preferentially degrades linear nuclear DNA while leaving circular mtDNA intact [100,101,102,103,104,105,106,107]. This approach has shown potential utility in forensic mtDNA workflows by reducing nuclear DNA interference during sequencing. In wildlife forensic studies involving *Panthera tigris* samples, ExoV treatment decreased ambiguous nucleotide calls associated with NUMT co-amplification, thereby improving mitochondrial sequence interpretation and taxonomic identification [108]. Although these findings support the forensic potential of enzymatic depletion strategies, further optimization and validation are required before routine implementation.

Table 2 summarizes the main mtDNA enrichment strategies reported in the literature, emphasizing their mechanistic foundations, advantages, limitations, and current relevance to forensic applications.

## 6. Sanger Sequencing Approaches in Mitochondrial DNA Analysis

### 6.1. Principles and Forensic Applications of Sanger Sequencing

Amplicon-based sequencing is a targeted approach in which specific genomic regions are selectively amplified prior to sequencing, traditionally using PCR [111] or enzymatic fragmentation [112]. This methodology depends on the design of primers that flank the regions of interest, thus enabling deep and focused coverage of the selected loci [12,113].

Historically, forensic analysis of mtDNA has relied on Sanger sequencing as a reference method [12]. This technique involves PCR amplification of defined target regions, followed by chain-termination sequencing using fluorescently labeled dideoxynucleotides and fragment separation via capillary electrophoresis [111,113].

As mentioned earlier, early applications included historical identifications, such as the Romanov family case, in which mtDNA sequencing confirmed maternal lineage relationships from skeletal remains [29,114]. Furthermore, this approach proved particularly valuable in forensic casework involving highly compromised human remains, where conventional morphological or STR-based analyses have been unsuccessful. Early forensic applications have demonstrated that mtDNA sequencing can generate reproducible profiles from skeletal remains up to 75 years old and support the identification of missing persons through comparison with maternal relatives [115]. Similarly, in a two-decade-old homicide investigation involving highly degraded and fragmented bones, mtDNA analysis combined with next-generation and Sanger sequencing enabled the identification of a human bone fragment among multiple nonhuman remains and supported kinship confirmation with putative parents [116].

### 6.2. Limitations of Sanger Sequencing

Despite its robustness and long-standing use in forensic genetics, Sanger sequencing is inherently constrained by limited read length and low analytical throughput, typically generating a single read of approximately 25–1200 bp/run [117,118,119]. These limitations are primarily associated with the reduced resolution of capillary electrophoresis for larger DNA fragments and the progressive decline in signal quality during ddNTP incorporation, both of which increase base-calling uncertainty in the terminal regions of the sequence [120,121]. Interpretation may be further complicated by the presence of homopolymeric C-stretches within the mitochondrial control region, which are particularly prone to polymerase slippage and sequencing artifacts. These regions can generate ambiguous base calling, alignment difficulties, and reduced reproducibility across sequencing platforms, thereby requiring careful prescreening and rigorous data interpretation [122,123,124].

Although Sanger sequencing remains a robust and well-established methodology, its low throughput and labor-intensive workflow limit its suitability for complete mitochondrial genome analysis [78,113,125]. These technical and operational constraints ultimately contributed to the transition toward NGS technologies in forensic mtDNA research and casework.

## 7. Next-Generation Sequencing Approaches in Mitochondrial DNA Analysis

### 7.1. Principles of NGS in Forensic mtDNA Analysis

NGS, also referred to as MPS, represents a major transition from single-target sequencing to high-throughput genomic analysis. Unlike Sanger sequencing, second-generation platforms simultaneously sequence millions of PCR-derived fragments, generally ranging from 150 to 300 bp, thereby enabling multiplexed analysis of numerous genomic targets in a single workflow [45,118,119]. In forensic genetics, NGS supports the concurrent characterization of autosomal, mitochondrial, and sex-linked markers [12,120].

The adoption of NGS has substantially improved forensic mtDNA analysis, particularly for degraded and low-template DNA samples. Whole mitogenome and control region sequencing studies have consistently demonstrated increased sensitivity, deeper coverage, improved heteroplasmy detection, and strong concordance with conventional Sanger sequencing approaches [121,122]. Methodological advances have also focused on mitigating challenges such as contamination, NUMTs interference, and low-frequency variant miscalling through optimized library preparation methods, balanced coverage strategies, and refined bioinformatic pipelines [123,124].

NGS has also expanded the utility of forensic mtDNA analysis in genealogy-based investigations and complex human identification scenarios involving degraded remains and trace evidence [125,126]. Whole-mitogenome NGS sequencing has been successfully applied to rootless hairs associated with the Romanov family [35], highly degraded forensic and archaeological remains [82,83], and fragmented forensic-type samples using overlapping-amplicon strategies [78]. MPS of mitochondrial DNA has strengthened humanitarian forensic investigations involving highly degraded human remains from armed conflicts, mass disasters, and missing persons cases. For example, large-scale identification programs associated with the Balkan conflicts, the World Trade Center disaster, and missing persons investigations have incorporated advanced mtDNA analysis to support maternal lineage reconstruction and kinship comparisons from degraded remains [126]. In addition, operational forensic programs, such as the California Department of Justice Missing Persons DNA Program, have implemented NGS-based mtDNA analysis to improve identification success rates from compromised samples [127].

### 7.2. Methodological Developments in NGS-Based mtDNA Forensic Workflows

Several commercially available forensic workflows illustrate these technological developments. Current MPS-based mtDNA assays typically rely on tiled short-amplicon strategies to maximize recovery from degraded material and achieve near-complete mitochondrial genome coverage [127,128,129,130,131,132,133,134,135,136,137]. Validation studies across multiple platforms have consistently reported high read depth, reliable reproducibility, and strong concordance with Sanger-derived haplotypes, even when working with highly compromised forensic specimens such as aged bones, teeth, bloodstains, and rootless hair shafts [123,129,130,131,132,133,134,135,136,137,138]. In comparison with traditional control-region sequencing, these approaches improve the discriminatory power by enabling full mitogenome recovery and more sensitive heteroplasmy detection [121,130,134].

### 7.3. Limitations and Operational Challenges of NGS

Despite these advantages, NGS-based mtDNA analysis presents important technical and operational limitations. PCR amplification remains necessary in most workflows, potentially introducing amplification bias, clonal redundancy, and uneven coverage across specific amplicons [135,137]. Increased analytical sensitivity also increases susceptibility to contamination and inhibitor effects [136]. Furthermore, the interpretation of low-level heteroplasmy and management of large sequencing datasets require extensive downstream bioinformatic analysis and standardized reporting practices [123,124,137]. Finally, the relatively high cost and infrastructure requirements of MPS workflows may limit their routine implementation in some forensic laboratories.

## 8. Third-Generation Sequencing in mtDNA Analysis

### 8.1. Long-Read Sequencing Technologies and Forensic Applications

Third-generation sequencing (TGS), also referred to as long-read sequencing, represents the latest advancement in sequencing technologies, enabling the real-time analysis of single DNA molecules with or without prior PCR amplification [128,129]. The principal platforms, Pacific Biosciences (PacBio) and Oxford Nanopore Technologies (ONT), differ in their underlying chemistries; however, both can generate read lengths that span kilobases to megabases [130,131,132,133].

Among TGS platforms, nanopore sequencing has gained the greatest traction in forensic research, with numerous studies evaluating its performance in STR, SNP, and mtDNA analyses [134,135,136,137,138,139]. Its key advantages include portability, which enables on-site analysis, and the ability to sequence the native DNA. Nanopore sequencing has demonstrated reliable STR and SNP genotyping, strong performance in microhaplotype analysis, ability to directly detect epigenetic modifications, and notable utility for mtDNA analysis and species identification [89,135,137]. Long-read sequencing additionally enables high-coverage full-length mitochondrial genome analysis with strong concordance to short-read amplicon-based methods [139].

Recent studies have demonstrated the potential of TGS to overcome several limitations associated with short-read sequencing. Long-range PCR and nanopore-based workflows have successfully generated continuous reads spanning the entire mitochondrial genome, thereby improving haplotype resolution and reducing interference from NUMTs [73], Dedicated bioinformatic pipelines have further enabled sensitive heteroplasmy detection and automated variant annotation, with some studies reporting accurate identification of heteroplasmic variants at frequencies as low as 5% [140]. Long-read sequencing has also shown promise in resolving mixed mtDNA samples and improving haplotype-level discrimination in complex forensic scenarios [141,142].

### 8.2. PCR-Free and Targeted Enrichment Strategies

An important advantage of TGS is the possibility of PCR-free enrichment. Restriction enzyme-based linearization approaches (using enzymes such as BamHI or PvuII) and CRISPR–Cas9 targeted sequencing methods, such as nanopore Cas9 Targeted Sequencing (nCATS), allow the selective enrichment of intact mtDNA molecules prior to sequencing [101,103,106,118,132,143,144,145]. By avoiding PCR amplification, these strategies reduce amplification bias and improve the characterization of large deletions, structural variations, heteroplasmy phasing, and full-length mitogenome recovery. Recent studies have applied nCATS workflows to complete human and nonhuman mitochondrial genome sequencing, as well as to the characterization of age-associated mitochondrial deletions [103,146,147]. Collectively, these investigations underscore the potential of PCR-free long-read enrichment approaches for high-resolution mtDNA analysis. However, to our knowledge, nCATS strategies have not yet been validated for routine forensic mtDNA casework and currently require relatively large DNA inputs, which may limit their applicability to highly degraded or low-template forensic samples.

### 8.3. Epigenetic Applications of Third-Generation Sequencing

Beyond sequence analysis, TGS technologies enable the direct detection of epigenetic modifications in native DNA molecules. Forensic epigenetics primarily leverages DNA methylation at CpG sites as a highly stable and informative biomarker for various forensic applications. According to Vidaki et al. [148] and Gerra et al. [149], specific epigenetic markers are essential in three main areas: tissue type identification, which utilizes cell-specific methylation patterns to distinguish between biological fluids; age estimation based on “epigenetic clocks” or age-correlated CpG sites that reflect the biological aging process; and differentiation of monozygotic twins, where stochastic epigenetic drift provides a unique molecular signature that cannot be captured by standard STR profiling. Emerging research highlights the potential of these markers in estimating the post-mortem interval (PMI) and characterizing an individual’s lifestyle or environmental exposure, although these applications still face significant challenges regarding environmental degradation and tissue-specific variability [149].

Nanopore sequencing enables the direct detection of epigenetic modifications by analyzing native DNA without requiring bisulfite treatment. In this context, Zeng et al. [89] identified and validated 3820 shared differentially methylated loci (DMLs) across six monozygotic twin pairs, encompassing CpG and non-CpG methylation sites. With >99.5% alignment efficiency and long read lengths (N50 > 13 kb), the study demonstrated the technical robustness of long-read nanopore sequencing for single-base resolution methylation profiling and proposed these loci as candidate biomarkers for forensic twin differentiation [89].

As summarized in Table 3, forensic mtDNA sequencing strategies differ in terms of their analytical resolution, maturity, and susceptibility to bias.

**Figure 2:**
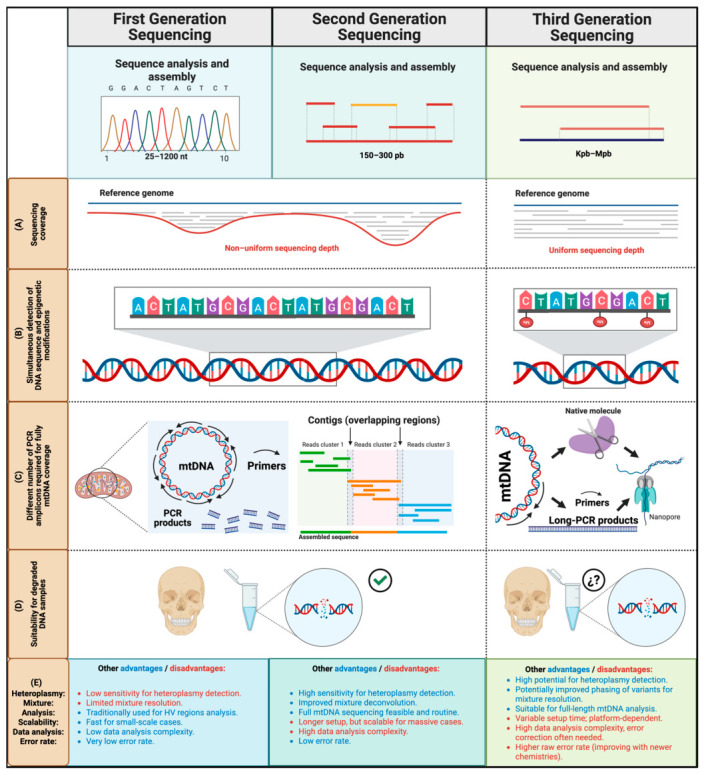
Key differences among first-, second-, and third-generation sequencing technologies in mitochondrial DNA (mtDNA) analysis. The figure summarizes the methodological distinctions and typical workflows across sequencing generations. Top: Representative workflows for each approach: first-generation (Sanger) sequencing illustrated by an electropherogram; second-generation sequencing showing short-read library preparation, high-throughput sequencing, and computational assembly; and third-generation sequencing depicting long-read, single-molecule sequencing, and assembly. (**A**) Coverage patterns: First- and second-generation methods generate variable fragment lengths and often yield uneven genome coverage, whereas third-generation technologies provide more uniform long-read coverage. (**B**) Epigenetic modification detection: Third-generation platforms can directly identify epigenetic marks in native DNA, whereas first- and second-generation approaches generally require additional chemical treatments or cannot directly detect these modifications. (**C**) Library preparation and amplification: First- and second-generation methods typically depend on multiple PCR amplicons for full mtDNA coverage, whereas third-generation approaches can sequence long DNA fragments with minimal amplification (e.g., enzymatic fragmentation or long-range PCR). (**D**) Forensic applicability: First- and second-generation technologies are well validated and widely implemented in forensic workflows, whereas third-generation sequencing remains under evaluation and is not yet broadly adopted for routine casework. (**E**) Additional advantages (blue) and disadvantages (red) associated with each sequencing generation method. Figure was created using BioRender.com.

Despite these advantages, the routine implementation of TGS in forensic genetics remains limited, with most studies to date focusing on developmental validation, proof-of-concept applications, and the adaptation of non-forensic protocols. Challenges include higher input DNA requirements, platform-specific error profiles, and the need for forensically validated bioinformatic pipelines and interpretation guidelines. Nevertheless, ongoing improvements in sequencing accuracy, library preparation methods, and analytical software continue to lower these barriers, positioning TGS as a promising tool for advanced forensic mtDNA analysis. Figure 2 summarizes the main differences between the sequencing technologies.

## 9. Limitations and Interpretative Challenges of Forensic mtDNA Analysis

Despite its utility in degraded and low-template samples, mtDNA analysis presents important biological and analytical limitations that constrain its value in forensic human identification. Unlike autosomal STR profiling, mtDNA is maternally inherited and lacks recombination, meaning that maternally related individuals frequently share identical haplotypes. Consequently, mtDNA evidence is primarily associative or exclusionary rather than individually identifying, and its discriminatory power remains lower than that of nuclear DNA typing [179].

Heteroplasmy further complicates interpretation because mtDNA variants may differ among tissues, across generations, and between maternal relatives. The increased sensitivity of MPS has also revealed low-frequency variants that may represent true biological signals, sequencing artifacts, contamination, or stochastic amplification effects, making the interpretation of thresholds an ongoing challenge in forensic standardization [11,21,180,181].

Another important limitation is the presence of NUMTs, which can be co-amplified or co-enriched during analysis and generate misleading mtDNA variants, particularly in degraded samples [182]. In addition, the high copy number of mtDNA increases susceptibility to contamination, requiring stringent laboratory controls, replicate analyses and phylogenetic consistency checks [11].

The evidentiary interpretation of mtDNA also depends heavily on population databases such as EMPOP, although population representation remains uneven across geographic regions, potentially affecting haplotype frequency estimates and likelihood ratio calculations [183,184]. Furthermore, post-mortem DNA damage may generate artifactual substitutions that mimic genuine variants, particularly in highly degraded remains analyzed using ultrasensitive sequencing technologies [185,186]. Overall, although mtDNA remains a powerful complementary tool in forensic genetics, its interpretation relies on lineage association and probabilistic inference rather than on definitive individual identification.

## 10. Decision-Making Frameworks for Forensic mtDNA Analysis

**Figure 3:**
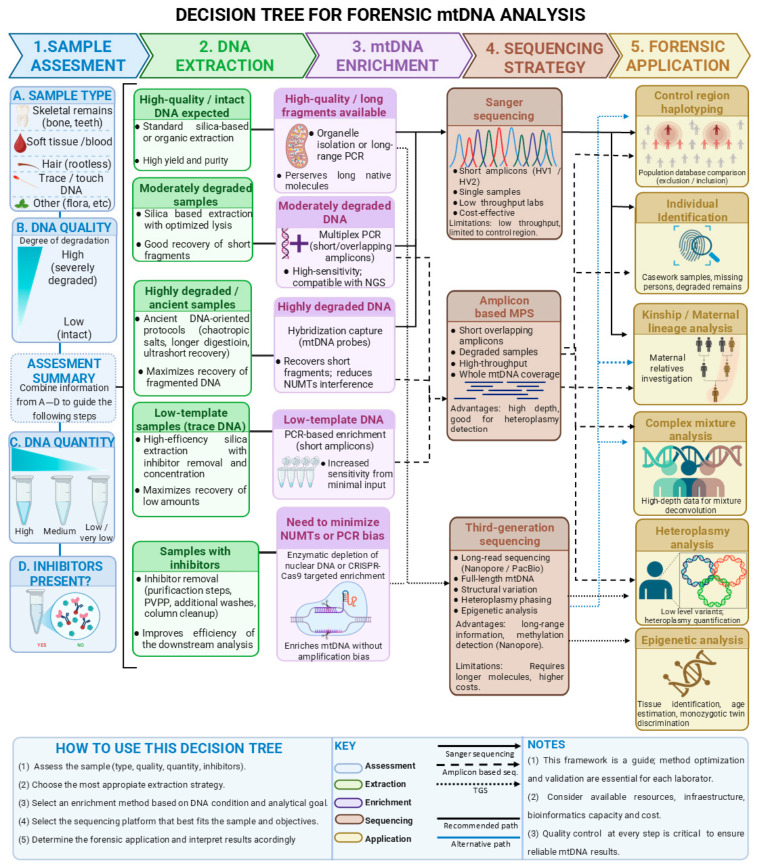
Decision tree summarizing the principal methodological approaches used in forensic mtDNA analysis according to sample preservation, DNA quality, analytical objectives, and sequencing strategy. The framework integrates DNA extraction methods, mtDNA enrichment approaches, and sequencing technologies, including Sanger sequencing, next-generation sequencing (NGS/MPS), and third-generation sequencing (TGS), highlighting their principal forensic applications, advantages, and technical limitations in human identification, degraded sample analysis, heteroplasmy detection, and epigenetic characterization of mtDNA. Figure was created using BioRender.com.

The selection of extraction, enrichment, and sequencing strategies in forensic mtDNA analysis depends on multiple analytical variables, including sample preservation, DNA degradation, quantity, and the intended forensic application. Figure 3 presents a decision-making framework that integrates sample assessment, DNA extraction, mtDNA enrichment, sequencing strategies, and their corresponding forensic applications.

The diagram illustrates how different analytical pathways may be selected according to the sample quality, DNA preservation, and forensic objectives. Recommended pathways correspond to sequencing approaches most commonly associated with specific forensic applications under current practice, such as Sanger sequencing for control-region haplotyping, targeted MPS workflows for individual identification, and third-generation sequencing for epigenetic applications. Alternative pathways indicate complementary applications in which a given sequencing strategy may still provide informative results depending on the sample condition or laboratory infrastructure. Overall, this framework highlights the progressive integration of extraction, enrichment, and sequencing methodologies in contemporary forensic mtDNA analysis.

## 11. Conclusions

Future progress in forensic applications will depend on coordinated advancements across the entire analytical workflow, including optimized DNA recovery strategies, efficient mtDNA enrichment, and robust sequencing techniques. Although many of the methods reviewed here have demonstrated feasibility in biomedical, population genetics, and ancient DNA research, only a limited subset has undergone formal forensic validation, according to established guidelines. Consequently, several approaches remain experimental or insufficiently standardized, with limited assessments of key parameters such as sensitivity, reproducibility, susceptibility to exogenous DNA interference, and interpretative robustness under forensic conditions.

As these methodologies mature, rigorous validation and standardization will be essential to ensure reproducibility, minimize bias, and support reliable interpretation. When integrated with well-curated population databases, standardized nomenclature, and transparent reporting frameworks, modern mtDNA sequencing has the potential to further enhance the resolution and evidentiary value of human identification and ancestry inference in forensic science.

## Figures and Tables

**Table 1:** Comparative overview of extraction approaches used in mitochondrial DNA forensic analyses.

Method Category	Principle	Advantages	Limitations	Best Suited Sample Types	Key References
Organic extraction (phenol–chloroform)	Organic phase separation following lysis and protein digestion	High DNA recovery	Toxic reagents, labor-intensive, low automation	Bone, teeth, soft tissues	[38,41,46,50,51,52,53]
Silica-based extraction	DNA adsorption to silica under chaotropic conditions	Efficient purification, inhibitor removal, suitable for degraded DNA	Possible loss of ultrashort fragments; limited input capacity	Bone, teeth, degraded skeletal remains	[37,39,40,41,54,55]
Magnetic bead–based extraction	Magnetic capture and purification of DNA-bound beads	Automation-compatible, scalable, reduced handling	Requires optimization for highly degraded DNA	Blood, hair shafts, degraded forensic samples	[37,44,48,54,56]
Hybrid workflows	Combined demineralization, optimized digestion, and silica/bead purification	Improved recovery of ultrashort fragments	More complex workflows and contamination control requirements	Burned remains, ancient bones, highly degraded skeletal material	[39,40,42,43,44,45,49,54,57]

**Table 2:** Comparative overview of mitochondrial DNA enrichment strategies employed in forensic genetics.

Method	Principle	Advantages	Limitations	Best Suited Sample Type	Forensic Validation Status	Key References
Plasmid miniprep–based selective extraction	Preferential isolation of circular mtDNA	Very high enrichment (>2000-fold)	Limited forensic validation	Moderately degraded samples	Emerging	[55]
PCR-based enrichment	Selective amplification of mtDNA regions	High sensitivity and broad forensic applicability	Amplification bias; heteroplasmy distortion	Low-template and degraded DNA	Established	[11,68,69,70,72,73,74,75,90,91]
Hybridization-based capture	Probe-based enrichment of mtDNA fragments	Effective for fragmented DNA; reduced NUMT interference	Costly and labor-intensive workflows	Degraded skeletal remains	Established	[82,84,85,86,87,94,95,96,97,98,99,109]
Enzymatic enrichment	Selective degradation of linear nuclear DNA	Reduces nuclear background	Requires intact circular mtDNA	Moderately degraded DNA	Experimental (non-forensic)	[100,101,102,103,104,105,106,107,108,110]

**Table 3:** Comparative overview of sequencing approaches used in forensic mitochondrial DNA.

Sequencing Category	Method/Approach	Principle	Main Strengths	Main Limitations	Most Suitable Samples	Validation Status	Key References
Amplicon-based sequencing	Sanger sequencing (control region)	PCR amplification followed by chain-termination sequencing	High accuracy; standardized interpretation	Low throughput; labor-intensive whole mitogenome analysis	Low to moderately degraded samples	Established	[29,111,114,115,116,123,125,150,151,152,153,154]
	Overlapping amplicon strategies	Multiple short amplicons covering mtDNA regions	Improved compatibility with degraded DNA	Primer-site bias; limited long-range resolution	Degraded forensic samples	Established	[155,156,157]
Next-generation sequencing (NGS/MPS)	Short-read massively parallel sequencing	Parallel sequencing of millions of short fragments	High throughput; sensitive heteroplasmy detection; whole mitogenome recovery	PCR bias; NUMT interference; complex bioinformatics	Low-template and degraded DNA	Established	[13,121,158,159,160,161,162]
	Targeted mtDNA MPS assays	PCR-based targeted enrichment coupled with MPS	Standardized forensic workflows; high sensitivity	Amplification bias; workflow dependency	Routine forensic casework	Established	[77,127,160,163,164,165,166,167,168,169,170,171,172,173,174]
	Hybridization capture + MPS	Probe-based enrichment prior to sequencing	Effective for highly degraded DNA; reduced NUMT interference	Costly and time-intensive workflows	Skeletal remains; ancient-like samples	Established	[82,84,87,96,99,139,175]
Long-read sequencing (TGS)	Oxford Nanopore Technologies (ONT)	Single-molecule nanopore sequencing	Full-length mtDNA analysis; heteroplasmy phasing	Higher raw error rates; limited forensic standardization	Moderately degraded to high-quality DNA	Emerging	[73,101,106,128,142]
	Pacific Biosciences HiFi sequencing	Single-molecule real-time long-read sequencing	High-accuracy long reads; uniform coverage	High cost and infrastructure requirements	High-quality DNA extracts	Emerging	[176,177]
	CRISPR/Cas9-assisted long-read enrichment	Targeted cleavage and enrichment of mtDNA	Facilitates full-length mtDNA recovery	Limited forensic implementation	High-quality DNA extracts	Experimental	[103,106,140,145,178]

## Data Availability

No new data were created or analyzed during this study. Data sharing is not applicable to this article.

## Abbreviations

The following abbreviations are used in this manuscript.

mtDNA	Mitochondrial DNA
NUMTs	Nuclear Mitochondrial DNA sequences
OXPHOS	Oxidative phosphorylation
SNV	Single Nucleotide Variants
INDELS	Insertion-Deletions
D-loop	Displacement Loop
HVR	Hypervariable Region
HID	Human Identification
rCRS	Revised Cambridge Reference Sequence
RSRS	Reconstructed Sapiens Reference Sequences
STR	Short Tandem Repeat
NGS	Next Generation Sequencing
MPS	Massively Parallel Sequencing
ExoV	Exonuclease V
UAS	Universal Analysis Software
TGS	Third-generation Sequencing
ONT	Oxford Nanopore Technologies
nCATS	Nanopore Cas-9 Targeted Sequencing
PMI	Post-mortem Interval
DML	Differentially Methylated Loci
LR	Likelihood Ratio
